# Internalized Nanoceria Modify the Radiation-Sensitivity Profile of MDA MB231 Breast Carcinoma Cells

**DOI:** 10.3390/biology10111148

**Published:** 2021-11-08

**Authors:** Emory Bibb, Noura Alajlan, Saad Alsuwailem, Benjamin Mitchell, Amy Brady, Muhammad Maqbool, Remo George

**Affiliations:** 1 Nuclear Medicine and Molecular Imaging Sciences Program, Department of Clinical and Diagnostic Sciences, School of Health Professions, University of Alabama at Birmingham, Birmingham, AL 35294, USA; ebibb43@uab.edu (E.B.); nouraaj1994@gmail.com (N.A.); suwailem@uab.edu (S.A.); bmitch23@uab.edu (B.M.); amybrady@uab.edu (A.B.); 2 Health Physics Program, Department of Clinical and Diagnostic Sciences, School of Health Professions, University of Alabama at Birmingham, Birmingham, AL 35294, USA; mmaqbool@uab.edu

**Keywords:** radiosensitivity, radiation protection, cerium oxide nanoparticles, MDA MB231 cells, macropinocytosis, macropinosomes, apoptosis, reactive oxygen species

## Abstract

**Simple Summary:**

Cerium oxide nanoparticles (nanoceria) influence its environment by donating or accepting electrons readily. We investigated nanoceria’s ability to modify the health of treatment-resistant breast cancer cell type, with various doses of potentially damaging radiation. We used electron microscopy to determine the presence and location of various amounts of nanoceria in cells. We also used imaging modalities such as confocal fluorescence and flow cytometry to study cell-health, cell-death, and amounts of potentially damaging unstable molecules called reactive oxygen species, all without or with different doses of radiation. Our results showed that nanoceria were taken up by a cell-drinking process called macropinocytosis, and then got segregated into large compartments called macropinosomes. There was an overall decrease in cell-death with increasing nanoparticle concentrations. This increase in cell-health resulted in a reduction of the reactive oxygen species at all tested radiation doses. Moreover, this effect appeared prominent at lower radiation doses compared to populations not treated with radiation or nanoparticles. In conclusion, our discovery shows that internalized nanoceria provide protection from radiation with a corresponding decrease in reactive oxygen species in this type of breast cancer cells and this property confers significant perils and opportunities when utilized in the context of cancer radiation therapy.

**Abstract:**

Owing to its unique redox properties, cerium oxide (nanoceria) nanoparticles have been shown to confer either radiosensitization or radioprotection to human cells. We investigated nanoceria’s ability to modify cellular health and reactive oxygen species (ROS) at various absorbed doses (Gray) of ionizing radiation in MDA-MB231 breast carcinoma cells. We used transmission electron microscopy to visualize the uptake and compartmental localization of nanoceria within cells at various treatment concentrations. The effects on apoptosis and other cellular health parameters were assessed using confocal fluorescence imaging and flow cytometry without and with various absorbed doses of ionizing radiation, along with intracellular ROS levels. Our results showed that nanoceria were taken up into cells mainly by macropinocytosis and segregated into concentration-dependent large aggregates in macropinosomes. Confocal imaging and flow cytometry data showed an overall decrease in apoptotic cell populations in proportion to increasing nanoparticle concentrations. This increase in cellular health was observed with a corresponding reduction in ROS at all tested absorbed doses. Moreover, this effect appeared pronounced at lower doses compared to unirradiated or untreated populations. In conclusion, internalized nanoceria confers radioprotection with a corresponding decrease in ROS in MDA-MB231 cells, and this property confers significant perils and opportunities when utilized in the context of radiotherapy.

## 1. Introduction

Ionizing radiation has been used quite early on in a variety of clinical areas since the discovery of X-rays and radioactivity in the late 1800s, including physiology, immunology, diagnostic, and therapeutic medicine [1]. X-rays and gamma rays are ionizing radiation due to their shorter wavelengths and high energies, that are capable of breaking chemical bonds, resulting in altered structures and functions of the cells, tissues, and organs that they penetrate [2]. However, the trillions of cells in our body are bombarded each second with ionizing radiation from the natural background [3]. Radiation responses in living systems are generally thought to follow a linear non-threshold model with little or no effects at low absorbed doses, while having serious health ramifications at higher doses [4]. The realm where stochastic effects merge into deterministic effects for diseases like cancer is still being debated, but there is a general scientific consensus around the fact that diagnostic levels of radiation below the regulatory limits are considered relatively safe, while significant protection needs to be afforded at higher levels of radiation [5]. The initial action of ionizing radiation is two-fold: direct ionization of cellular structures, especially DNA, and the radiolysis of water, which accounts for 70% or more of the total cell mass [6]. The former results in chromosomal aberrations, DNA damage, mutagenesis, and carcinogenesis, while the latter leads to the production of a variety of reactive oxygen species and the amplification of free radicals, causing structural damage to biomolecules in their vicinity, including proteins, lipids, and nucleic acids [7]. Cells have natural protective mechanisms in place with DNA repair mechanisms and free-radical scavengers to counter the effects of radiation at lower absorbed doses, but this protective apparatus breaks down when overwhelmed by radiation [8]. This is significant in the context of nuclear medicine and radiation therapy, and even more so at comparatively lower therapeutic doses, where significant detrimental bystander effects can occur mainly due to the intercellular signaling propagated from the site of irradiation to surrounding healthy tissues [9]. A variety of agents have been investigated as radioprotectants [10,11,12,13,14,15,16,17,18,19], but the only one that made it into the clinics for approved use in patients is Amifostine (WR-2721), with a very short half-life in serum [20]. Radiosensitizers, on the other hand, are agents that enhance the therapeutic ratio of radiation therapy by enhancing DNA damage, free-radical production, or both when combined with radiation, while not damaging the normal cells in the process. A few potential clinical radiosensitizers are currently being used in patient trials [21,22,23,24,25,26,27], in the context of a historic backdrop of many others, ultimately resulting in limited clinical efficacy and increased toxicity to normal tissues [28].

Nanomedicine has shown promise in improving the therapeutic outcomes due to a better tumor targeting and its increased permeability and retention effect, though issues with biocompatibility and poor uptake in the target tissues have been major clinical challenges [29]. Metallic nanoparticles have shown promise as radiosensitizers by inducing the production of free radicals following irradiation, leading to dose-enhancing bystander effects [30]. Cerium oxide (CeO_2_) nanoparticles (NPs), which belong to the rare earth lanthanide series of transition metals, have been shown to possess unique free-radical scavenging properties by modulating its ratio of Ce^3+^ to Ce^4+^ ions [31].

Previous studies have reported some paradoxical roles of nanoceria, with some studies showing a protective effect from free radical damage [32,33], while others have reported an increased induction of oxidative stress [34,35]. In cancer cells, cerium oxide nanoparticles have been shown to confer some wide-ranging effects, from anti-invasion [36] to radiosensitization [37] and radioprotection [38]. Reported effects of nanoceria on health and ecology also continued to range widely from no detrimental effects [39,40] to serious health effects including lung [41] and kidney [42] damage. Ultimately, the method and purpose of the clinical use of nanoceria in translation medicine may depend on local factors such as pH, that may determine the overall efficacy, cytotoxicity, and radiobiological properties of this unique rare-earth element.

Our previous studies have shown that pure cerium oxide nanoparticles can localize in triple-negative breast cancer cells without cytotoxicity [43]. In our present study, we hypothesized that pure cerium oxide nanoparticles will modify the radiosensitivity profile of triple-negative breast cancer cells from within their intracellular location. This hypothesis was tested in MDA MD231 cells using transmission electron microscopy, confocal imaging, flow cytometry, cell survival, and reactive oxygen species assays coupled with detailed statistical analyses of the results.

## 2. Materials and Methods

### 2.1. Culture of MDA MB231 Cells

The breast adenocarcinoma cell line MDA MB231 was obtained from the American Type Culture Collection (ATCC, Rockville, MD; product number: HTB-26). The cells were maintained in Leibovitz’s L-15 media (Corning, Manassas, VA, USA) supplemented with 10% fetal bovine serum (FBS) (HyClone, GE Health Care Life Sciences, Marlborough, MA, USA), and 1% penicillin-streptomycin (Gibco, Gaithersburg, MD, USA) in a humidified incubator at 37 °C. The cells were passaged every third day at approximately 80% confluency using a standard trypsin-EDTA (0.25%:0.2%) protocol (Gibco, Gaithersburg, MD, USA).

### 2.2. Cellular Uptake and Compartmental Localization of Nanoparticles

The internalization and compartmental localization of CeO_2_ nanoparticles (30% colloidal suspension in water produced by Alfa Aesar, Ward Hill, MA) were assessed using electron microscopy. We had previously characterized the CeO_2_ nanoparticles with Raman spectroscopy, which revealed a main band around 460 cm^−1^ corresponding to pure ceria and triply degenerate symmetric breathing F_2g_ mode characteristic of the stretching vibration of Ce-O in the O_h_ point group within the fluorite-type cubic crystal structure of CeO_2_ [43]. MDA MB231 cells were seeded overnight in a 24-well plate at a density of 1 × 10^5^ cells/well. The cells were treated with either 0, 25, 50, 100, or 200 μg/mL of CeO_2_ NPs in 2 mL of L-15 complete media for 72 h. After removing the media, the cells were fixed in a 3% glutaraldehyde and a 1% paraformaldehyde in 0.1 M cacodylate buffer followed by post fixation in 1% osmium tetroxide in 0.1 M caco buffer. All the samples were then dehydrated to 100% ethanol followed by three changes in propylene oxide and overnight infiltration in a solution containing 50% propylene oxide and 50% Epon-812 resin. The samples were then embedded in fresh Epon-812. Thin sections were taken using a Leica Ultracut-6 ultramicrotome (Leica Microsystems, Buffalo Grove, IL, USA) and were contrasted with uranyl acetate and Reynold’s lead citrate. Following the sample preparation, images were obtained using a FEI Tecnai Spirit electron microscope (FEI Company, Hillsboro, OR, USA) equipped with an AMT-Biosprint digital camera (AMT, Woburn, MA, USA). For imaging the CeO_2_ nanoparticles (10% colloidal suspension in water produced by Alfa Aesar, Ward Hill, MA, USA) without cells, a 7 µL suspension was placed on carbon-stabilized formvar grids for 60 s followed by the wicking away of excess liquid. Grids were placed into the microscope and imaged.

### 2.3. Determination of the Effect of CeO_2_ Nanoparticles on Native Cellular Heath and Redox Systems

The effect of CeO_2_ nanoparticles on apoptosis in MDA MB231 cells was visualized using confocal fluorescence microscopy, and the percentage of viable, early, and late apoptotic and necrotic cells was determined using Annexin-V-FITC (AV) and Propidium Iodide (PI) flow cytometry, along with flow cytometry analyses of intracellular reactive oxygen species (ROS) levels using the peroxide-dependent oxidation of dihydrorhodamine 123 (DHR123) to fluorescent rhodamine 123. For the imaging apoptosis and the flow cytometry analyses of cellular health on unirradiated cells, MDA MB231 cells were seeded overnight in a 24-well plate at a density of 1 × 10^5^ cells/well. The cells were treated with either 0, 25, 50, 100, or 200 μg/mL of CeO_2_ NPs in 2 mL of L-15 complete media for 72 h. The cells not treated with CeO_2_ NPs were treated with 10 µM cisplatin separately. The attached and floating cells were collected by centrifugation and resuspended in 100 μL of a binding buffer containing 5 μL AV and 5 μL PI according to the manufacturer’s instructions. After the incubation period (15 min at room temperature), the cells were centrifuged, and the pellet was resuspended in 200 μL of a binding buffer. For the confocal imaging of apoptotic cells, 10 µL of the sample was placed under a coverslip on a slide, sealed with nail polish, and the imaging was performed using an inverted Nikon A1R-HD25 equipped with a 60X apochromat oil-immersion objective (Nikon Corp., Tokyo, Japan). For the flow cytometric analyses of cellular health, the cell suspensions were stored on ice, and a flow cytometry analysis of the AV/PI was performed within one hour. The fluorescence intensities (green BL1-H and red YL2-H) were measured using the flow cytometry. In each sample, an average of 5000 cells were recorded (gated to exclude cell debris), and the percentages of viable (AV^−^/PI^−^), early apoptotic (AV^+^/PI^−^), apoptotic and necrotic (AV^+^/PI^+^), and already dead (AV^−^/PI^+^) cells were analyzed with FlowJo. For determining the intracellular levels of reactive oxygen species, MDA MB231 cells were seeded overnight in a 24-well plate at a density of 2 × 10^5^ cells/well. The cells were treated with either 0, 25, 50, 100, or 200 μg/mL of CeO_2_ nanoparticles in 2 mL of L-15 complete media for 72 h. Adherent cells were harvested by trypsinization and not incubated or incubated in 500 μL of a medium containing 10 μM DHR for 30 min at 37 °C in the dark. The cells were then washed twice in 1X PBS, and the DHR green fluorescence was analyzed by flow-cytometry at FL1-H channel using an excitation wavelength of 488 nm and an emission of 530/30. The Median fluorescence intensity (MFI) was assessed after correcting for autofluorescence.

### 2.4. Determination of the Effect of CeO_2_ Nanoparticles on Cellular Health following Irradiation with Ionizing Radiation

The effect on cellular health following treatments with various concentrations of CeO_2_ nanoparticles in MDA MB231 cells and the irradiation with varying absorbed doses of ionizing radiation was examined by assessing the percentage of viable, early, and late apoptotic and necrotic cells using Annexin-V-FITC (AV) and Propidium Iodide (PI) flow cytometry. MDA MB231 cells were seeded overnight in 24-well plates at a density of 1 × 10^5^ cells/well. The cells were treated with either 0, 25, 50, 100, or 200 μg/mL of CeO_2_ NPs in 2 mL of L-15 complete media for 48 h. The cells not treated with CeO_2_ nanoparticles were treated separately with 10 µM cisplatin in another well. Following the incubation, the sample plates were not irradiated or irradiated with 0.1, 1, or 10 Gy ionizing photon radiation using an X-RAD 320 irradiator (Precision, North Bramford, CT, USA) equipped with a pencil dosimeter (PTW Unidose E, Freiburg, Germany). The sample plate with a polystyrene lid was placed at 50 cm source-to-specimen distance, and the pencil dosimeter was placed on the platform adjacent to the plate. A similar plate lid was also placed on the dosimeter to account for any shielding effects. The tube settings were at 320 kVp and 12 mAs, with the dose rate recorded at 375.8 mGy/min. The samples were irradiated for the appropriate absorbed dose amounts and were then returned to the incubator. Following incubation for 24 hrs., all attached and floating cells were collected by centrifugation, labeled with AV/PI, and the percentages of viable (AV^−^/PI^−^), early apoptotic (AV^+^/PI^−^), apoptotic and necrotic (AV^+^/PI^+^), and already dead (AV^−^/PI^+^) cells were analyzed as described previously.

### 2.5. Determination of the Effect of CeO_2_ Nanoparticles on Intracellular Reactive Oxygen Species Levels following Irradiation with Ionizing Radiation

The effect of various concentrations of CeO_2_ nanoparticles on the generation of reactive oxygen species in MDA MB231 cells following irradiation with varying absorbed doses of ionizing radiation was examined by measuring intracellular rhodamine 123 fluorescence levels using flow cytometry. MDA MB231 cells were seeded overnight in a 24-well plate at a density of 2 × 10^5^ cells/well. The cells were treated with either 0, 25, 50, 100, or 200 μg/mL of CeO_2_ nanoparticles in 2 mL of L-15 complete media for 48 h. The sample plates were not irradiated or irradiated with 0.1, 1, or 10 Gy ionizing photon radiation as described before and then returned to the incubator for another 24 h. Following the incubation, the cells were either not labeled or labeled with DHR123, and the MFI was measured using flow cytometry as described previously.

### 2.6. Statistical Analyses

At least three experiments were done for each experiment. The bar graphs indicate the averages of the three experiments, and the error bars represent the standard deviation between experiments. The statistical significances between treatments under test conditions were compared using the Prism 8 software (Graphpad, San Diego, CA, USA) (supplementary files). The *p*-values were determined using a one-way or two-way analysis of variance (ANOVA) and Tukey’s post-hoc pairwise multiple comparison two-way ANOVA for each *n*, and considered significant for *p* < 0.05.

## 3. Results

### 3.1. CeO_2_ NPs Localize within Cellular Compartments in MDA MB231 Cells

**Figure 1:**
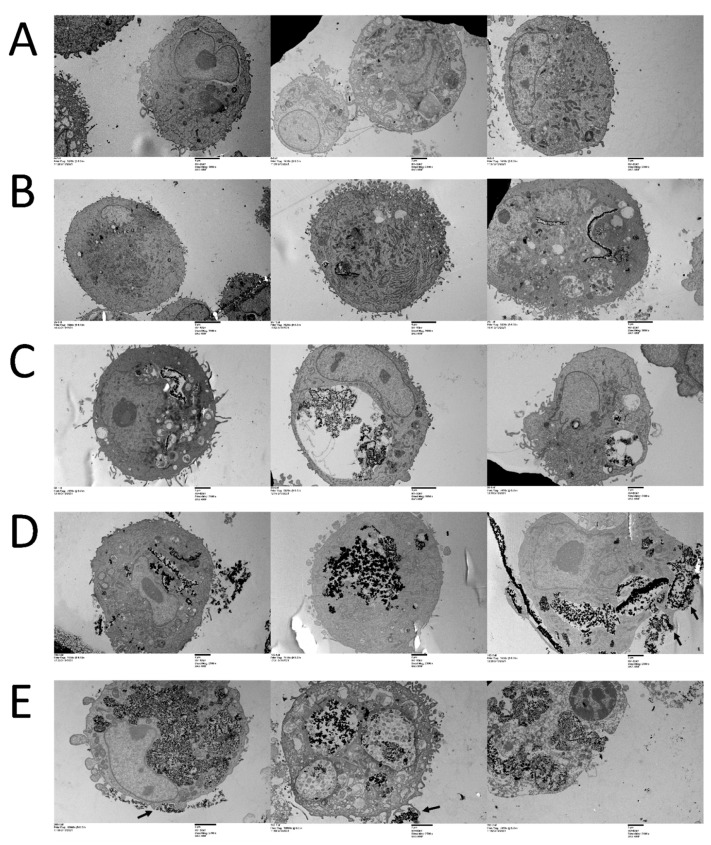
Compartmental visualization of CeO_2_ nanoparticles in MDA MB231 breast carcinoma cells. Random representative image samples of cells treated with (**A**) 0, (**B**) 25, (**C**) 50, (**D**) 100, or (**E**) 200µg mL^−1^ of CeO_2_ nanoparticles visualized under transmission electron microscopy.

We first evaluated the ability of MDA MB231 cells to take up the CeO_2_ nanoparticles and the precise site of localization within the cellular compartments. Transmission electron microscopy was performed on fixed, thin section embedded samples treated with 0, 25, 50, 100, or 200 µg mL^−1^ of CeO_2_ nanoparticles for 72 h (Figure 1). The treated cells demonstrated a copious uptake of nanoparticles (Figure 1B–E), while no similar uptake was noted in untreated cells (Figure 1A). The uptake appeared as the aggregates within the cells and the amount of these aggregates increased with increasing treatment concentrations of the nanoparticles. The nanoaggregates appeared to be mainly concentrated in walled structures occupying increasingly larger portions of the cell in proportion to the CeO_2_ NP treatment concentrations. Cell surface protrusions and invaginations enclosing CeO_2_ NP were also noted (Figure 1D,E, right panels, arrows). The nucleus, mitochondria, and cytoplasm in general appeared to be devoid of nanoaggregates.

### 3.2. CeO_2_ NPs Modulate Cellular Health and Reactive Oxygen Species in a Concentration-Dependent Manner in MDA MB231 Cells

**Figure 2:**
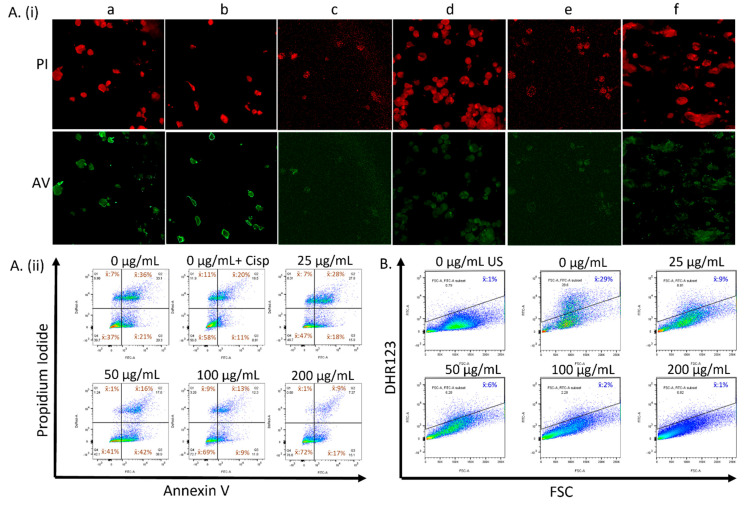
Concentration-dependent modulation of cellular health and reactive oxygen species by CeO_2_ nanoparticles in MDA MB231 breast carcinoma cells. (**A**). (**i**) Comparison confocal fluorescence images of cells labeled with propidium iodide (top panel) and Annexin V (bottom panel) following treatment with (**a**) 0, (**b**) 0 + Cisplatin, (**c**) 25, (**d**) 50, (**e**) 100, (**f**) 200 µg mL^−1^ CeO_2_ nanoparticles. (**A**). (**ii**) Four-quadrant gating of viable (AV^−^/PI^−^), early apoptotic (AV^+^/PI^−^), apoptotic and necrotic (AV^+^/PI^+^), and already dead (AV^−^/PI^+^) cells not treated or treated with cisplatin, 25, 50, 100, or 200 µg mL^−1^ nanoceria, analyzed for green fluorescence (Annexin V, FL1-A) and red fluorescence (Propidium Iodide, FL2-A). (**B**). Flow cytometric profiles of cells not labeled or labeled with 10 μM DHR123 following treatment with 0, 25, 50, 100, or 200 µg mL^−1^ nanoceria.

To investigate the effect of CeO_2_ NPs on cell death and on the native redox systems, MDA MB231 cells were either not treated or treated with 0, 25, 50, 100, or 200 µg/mL CeO_2_ NPs and labeled with either Annexin V/Propidium iodide (AV/PI) or 10 μM DHR123 (Figure 2). Cells displaying green and red fluorescence were visualized using confocal microscopy (Figure 2A(i)) and were quantified using flow cytometry at 525 nm (Annexin V, FL1-A) and 575 nm (Propidium Iodide, FL2-A) (Figure 2A(ii)). Cells gated for the FITC (525 nm) channel were used to assess the level of rhodamine 123 in cells not labeled or labeled with DHR123 (Figure 2B). Cells not treated with CeO_2_ NPs were treated separately with 10 µM cisplatin in another experiment (0 + Cip) and assessed for AV/PI localization (Figure 2A(ib,ii) top middle panel). Confocal images showed red fluorescence in all samples (Figure 2A(i) upper panel), while intense green fluorescence (Figure 2A(i) lower panel) was noted mostly in the cells not treated with nanoparticles or cisplatin (Figure 2A(ia)), and in the cells treated with cisplatin (Figure 2A(ib)).

The flow cytometry gated profiles of AV/PI—labeled cells that were treated with 0, 25, 50, 100, 200 µg/mL CeO_2_ NP, or 10 µM Cisplatin showed the following mean cell populations, represented in Table 1. In comparison to the untreated control, highly significant increases were noted in the AV^−^/PI^−^ population of the cells treated with 100 and 200 µg/mL CeO_2_ NP (*p* < 0.0001), even though no such significance was noted with 25 and 50 µg/mL CeO_2_ NP-treated cells, despite having comparatively higher values (Figure S2i FITC-A^−^, DsRed-A^−^ group). Also while comparing the untreated control, a concentration-depended progressive statistically significant decrease in values was noted for AV^+^/PI^+^ cells that were treated with 50 (*p* < 0.05), 100 (*p* < 0.005), or 200 (*p* < 0.0005) µg/mL CeO_2_ NP (Figure S2i FITC-A+, DsRed-A+ group), while no such significance was noted for the decreased values in the AV^+^/PI^−^ group (Figure S2i FITC-A+, DsRed-A- group). A decrease in AV^−^/PI^+^ populations was observed in cells treated with 50 and 200 µg/mL CeO_2_ NP in comparison to untreated controls; however, the changes to these populations across all samples were not found to be statistically significant (Figure S2i FITC-A^−^, DsRed-A^+^ group). There were also no statistically significant changes noted for AV^+^/PI^−^, AV^+^/PI^+^, and AV^−^/PI^+^ populations in the cisplatin-treated samples (Figure S2).

The flow cytometry gated profiles of DHR123-labeled cells expressing rhodamine 123 that were treated with 0, 25, 50, 100, or 200 µg/mL CeO_2_ NP showed 29%, 9%, 6%, 2%, and 1% mean cell populations, respectively. The progressive decrease in the cell populations was proportional to the increasing CeO_2_ NP concentration and was highly significant across all samples (*p* < 0.0001) when compared to the untreated ones. A significant decrease in the measured cell populations was also noted for samples treated with a higher concentration of CeO_2_ NPs in comparison to the ones treated with lower concentrations (*p* < 0.05 to 0.005) (Figure S2ii).

### 3.3. Concentration-Dependent Modulation of Cellular Health by CeO_2_ NPs in MDA MB231 Cells following Irradiation

To investigate the effect of CeO_2_ NPs on cellular health following irradiation with ionizing radiation, MDA MB231 cells were either not treated or treated with increasing concentrations of CeO_2_ NPs or cisplatin, irradiated with 0.1, 1, or 10 Gy, and then labeled with Annexin V/Propidium iodide. The flow cytometry gated profiles of AV/PI—labeled cells that were treated with 0, 0 + cisplatin, 25, 50, 100, or 200 µg/mL CeO_2_ NP showed the following mean cell populations after 0.1 Gy (Table 2), 1 Gy (Table 3), or 10 Gy (Table 4) irradiation.

**Figure 3:**
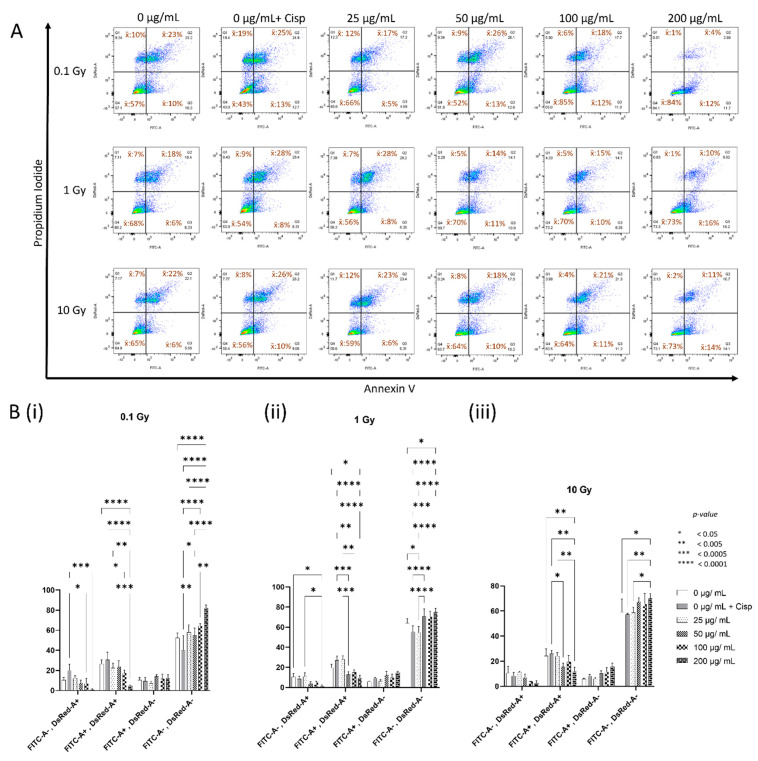
Concentration-dependent modulation of cellular health by CeO_2_ nanoparticles in MDA MB231 breast carcinoma cells following irradiation with ionizing radiation. (**A**). Four-quadrant gating of viable (AV^−^/PI^−^), early apoptotic (AV^+^/PI^−^), apoptotic and necrotic (AV^+^/PI^+^), and already dead (AV^−^/PI^+^) cells not treated or treated with cisplatin, 25, 50, 100, or 200 µg mL^−1^ nanoceria, analyzed for green fluorescence (Annexin V, FL1-A) and red fluorescence (Propidium Iodide, FL2-A) following 0.1, 1, or 10 Gy irradiation. (**B**). A two-way ANOVA was used to compare the background-subtracted median fluorescent intensities of different treatment samples within each FL1A/FL2A gated quadrant population irradiated with (**i**) 0.1 Gy, (**ii**) 1 Gy, or (**iii**) 10 Gy ionizing radiation.

A two-way ANOVA of all the samples (Figure 3B) showed that for samples irradiated with 0.1 Gy (Figure 3B(i)), an overall increase in the AV^−^/PI^−^ population was noted across all nanoparticle-treated samples, which was highly significant for 200 µg/mL (*p* < 0.0001) when compared to that of untreated irradiated negative controls. All nanoparticle-treated samples showed a significant increase in AV^−^/PI^−^ populations when compared to that of the cisplatin-treated irradiated positive controls (*p* < 0.05–0.0001). A corresponding nanoparticle concentration-dependent, overall decrease in the AV^+^/PI^+^ and AV^−^/PI^+^ populations was noted for all samples in comparison to the untreated negative controls, with the 200 µg/mL treatment group showing the most significant change for the AV^+^/PI^+^ population (*p* < 0.0001). Cisplatin-positive controls in the AV^+^/PI^+^ and AV^−^/PI^+^ groups showed an overall increase in their populations. The samples treated with 100 and 200 µg/mL nanoparticles showed the most significant decrease in AV^+^/PI^+^ and AV^−^/PI^+^ populations when compared to that of the cisplatin-treated irradiated positive control (*p* < 0.05–0.0001). No significant changes were noted for AV^+^/PI^−^ samples in this irradiation group.

Similar analyses of samples irradiated with 1 Gy (Figure 3B(ii)) showed an overall increase in the AV^−^/PI^−^ population for 50, 100, and 200 µg/mL nanoparticle-treated samples and was significant for the 200 µg/mL treatment (*p* < 0.05) when compared to that of the untreated irradiated negative controls. All nanoparticle-treated samples, except for 25 µg/mL, showed a highly significant increase in AV^−^/PI^−^ populations when compared to that of the cisplatin-treated irradiated positive controls (*p* < 0.0005–0.0001). A corresponding nanoparticle concentration-dependent, overall decrease in the AV^+^/PI^+^ and AV^−^/PI^+^ populations were noted for all samples in comparison to the untreated negative controls, except for the cisplatin-treated AV^+^/PI^+^ group, and this decrease was significant for the 200 µg/mL treatment (*p* < 0.05). All nanoparticle-treated samples, except for that of 25 µg/mL, showed a concentration-dependent significant decrease in AV^+^/PI^+^ populations when compared to that of the cisplatin-treated irradiated positive controls (*p* < 0.005–0.0001); however, no such significance was noted for the decrease in the AV^−^/PI^+^ population. Moreover, no significant changes were seen for AV^+^/PI^−^ samples in this group.

In comparing 10 Gy irradiated samples (Figure 3A, lower panel, and Figure 3B(iii)), an overall increase in the AV^−^/PI^−^ population was noted for 50, 100, and 200 µg/mL nanoparticle-treated samples in comparison to that of the untreated irradiated negative controls. This was found to be significant for the 200 µg/mL treatment samples (*p* < 0.05), similarly to other radiation doses for this population. The AV^−^/PI^−^ population across all irradiated samples significantly decreased with increasing radiation doses in this 200 µg/mL treatment group, while the other nanoparticle-treated groups mostly did not show any significant changes (Figure S3). A slight decrease was noted for the cisplatin-treated irradiated AV^−^/PI^−^ population (Figure 3B(iii)); however, this was not significant, despite an accentuated difference (*p* < 0.005) with the 200 µg/mL treatment samples. Despite this decrease in the AV^−^/PI^−^ samples for the cisplatin-treated group seen across all radiation doses, a significantly higher percentage was seen at higher doses (Figure S3, 0 µg/mL + Cisp). An overall decrease in the AV^+^/PI^+^ and AV^−^/PI^+^ populations was noted for all nanoparticle-treated samples in a concentration-dependent manner, in comparison to the untreated negative controls. This decrease was significant for the 200 µg/mL treatment samples in the AV^+^/PI^+^ population (*p* < 0.005) when compared to the irradiated negative and positive controls. An overall concentration-dependent increase in the AV^+^/PI^−^ population was also seen, but the changes noted in this group and in the AV^−^/PI^+^ population were not found to be significant.

### 3.4. Concentration-Dependent Modulation of Reactive Oxygen Species by CeO_2_ NPs in MDA MB231 Cells following Irradiation

To study the effect of CeO_2_ NPs in modulating the reactive oxygen species in cells following irradiation with ionizing radiation, MDA MB231 cells were either not treated or treated with increasing concentrations of CeO_2_ NPs, irradiated with 0.1, 1, or 10 Gy, and then labeled with DHR123. The flow cytometry gated profiles of rhodamine123-expressing cells that were treated with 0 (unlabeled, irradiated), 0, 25, 50, 100, or 200 µg/mL CeO_2_ NP showed the following background corrected mean cell populations after 0.1 Gy, 1 Gy, or 10 Gy irradiation, as represented in Table 5.

**Figure 4:**
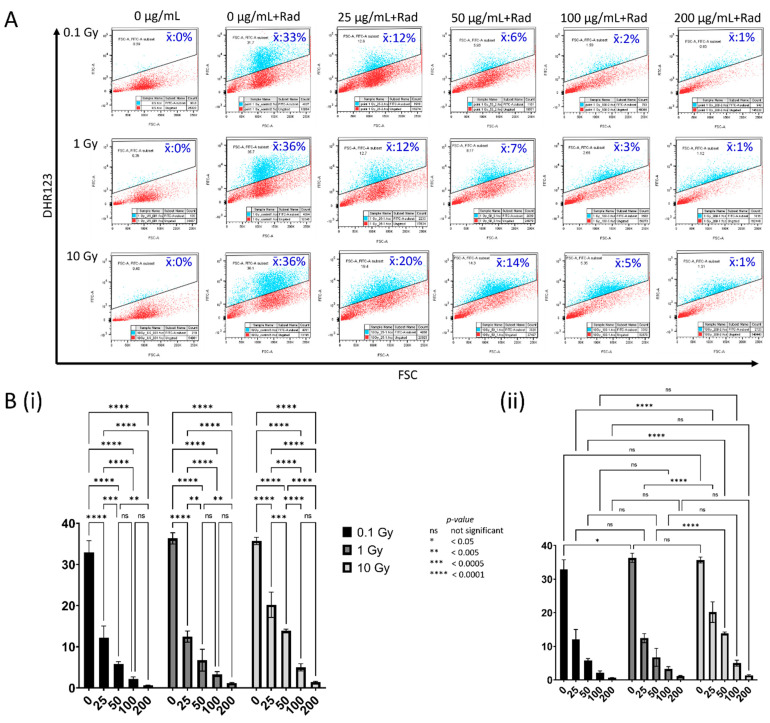
Concentration-dependent modulation of reactive oxygen species by CeO_2_ nanoparticles in MDA MB231 breast carcinoma cells following irradiation with ionizing radiation. (**A**). Flow cytometric profiles of 10 μM DHR123 labeled cells, not irradiated or irradiated with 0.1, 1, and 10 Gy ionizing radiation following treatment with 0, 25, 50, 100, or 200 µg mL^−1^ nanoceria. (**B**). A two-way ANOVA compares background-subtracted median fluorescent intensities in the FL1 subpopulations (**i**) within and (**ii**) in-between treated, irradiated sample groups.

A two-way ANOVA (Figure 4B) showed that for all samples irradiated with 0.1, 1, or 10 Gy, there was an overall highly significant decrease in the fluorescent gated population across all nanoparticle-treated samples (*p* < 0.0001) when compared to that of the untreated irradiated controls (Figure 4B(i)). This decrease was proportional to the increasing treatment concentration of the nanoparticles, with significant reductions noted between most of the treatments for each radiation dose. An inter-dose comparison of the results (Figure 4B(ii)) showed that there was a significant increase (*p* < 0.05) in fluorescence in the cells not treated with nanoparticles at 1 Gy when compared to 0.1 Gy, without any significant increase at 10 Gy. Incidentally, a corresponding finding was also observed for the AV/PI—labeled untreated irradiated cells, which showed a significant increase in the healthy population (*p* < 0.05) at 1 Gy when compared to that of 0.1 Gy, without any significant increase at 10 Gy (Figure S3, 0 µg/mL). Moreover, the cells treated with nanoparticles generally showed a significant reduction in fluorescence at all absorbed doses and treatment concentrations; however, a significantly higher fluorescence was noted for the 25 and 50 µg/mL treatments (*p* < 0.0001) at 10 Gy in comparison to similar treatments at other absorbed doses.

## 4. Discussion

In this study, we describe a systematic approach to track the uptake, localization, and modulation of the radiosensitivity of MDA MD231 breast carcinoma cells by unmodified cerium oxide nanoparticles. Studies over the past years have demonstrated the ability of high z number materials like gold and silver nanoparticles to absorb, scatter, and emit radiation energy, thereby acting as radiosensitizers [44,45,46,47,48,49]. There has been conflicting literature about cerium oxide nanoparticles’ ability to induce radiosensitization, despite being a metal with a high z number, with some studies showing radiosensitizing abilities [37,50,51,52], including in breast cancer [53], while others reporting it as a radioprotector [54,55,56]. Moreover, reports on the subcellular distribution of unmodified CeO_2_ nanoparticles also varied widely based on the type of cells: in neuronal stem cells, they were predominantly localized in membrane-bound structures and, to a lesser extent, were found free in the cytoplasm, with none detected in the nucleus or other structures [57]; in gastric cancer cells, they were reported to be localized in lysosomes and in no other parts of the cell [58]; in human keratinocyte cells, they were localized in mitochondria, lysosomes, and endoplasmic reticulum, as well as being abundant in the cytoplasm and the nucleus [59]; however, in bone marrow-derived macrophages, the CeO_2_ nanoparticles entered the nucleus and were found to disrupt the integrity of the cell membrane and organelles [60]. Moreover, it has been reported that the subcellular location of the nanoceria affected their cytotoxicity profiles, with minimal toxicity observed when localized in the cytoplasm and exhibiting significant cytotoxicity when present in the lysosomal compartment [35].

To precisely determine the concentration-dependent compartmental localization of CeO_2_ nanoparticles within the MDA MB231 cells, we performed transmission electron microscopy on cells treated with 0, 25, 50, 100, or 200 µg mL^−1^ of nanoparticles for 72 h (Figure 1). Compared to untreated controls, copious amounts of nanoparticles were observed inside the NP-treated cells. The cells appear to concentrate the NPs into aggregates in comparison to the uniformly distributed NPs in the treatment colloid solution (Figure S1). The concentration of these aggregates increased with increasing treatment concentrations of the nanoparticles. The nanoaggregates appeared to segregate into vesicle-like structures with increasing size to accommodate larger treatment concentrations. Plasma membrane protrusions enveloping the nanoparticles were observed on the cell surface, indicating a possible mechanism for taking up the metal oxide nanoparticles into the cells (Figure 1D,E, arrows). It is known that cells can create large membrane ruffles by cytoskeleton rearrangement, which are used to engulf nanomaterial-sized particles into cells [61]. These protrusions fold back and fuse with the plasma membrane to form large vesicles enclosing the nanoparticles, which then bud off to form macropinosomes containing the nanoparticle aggregates. The features observed here indicate that the major process of uptake of CeO_2_ nanoparticles in MDA MB231 cells is macropinocytosis. No significant presence of the nanoparticles was noted in any of the cellular organelles, including the nucleus, mitochondria, and general cytoplasm. To our knowledge, this is the first detailed report of the compartmental localization of CeO_2_ nanoparticles in MDA MB231 cells and the visualization of the concentration-dependent uptake of nanoparticles in these cells.

Our studies on the effects of CeO_2_ NPs to modify cellular health in MDA MB231 carcinoma cells involved both confocal imaging and flow cytometric analyses of cells taking up Annexin V (AV) and Propidium Iodide (PI) fluorophores. Confocal imaging demonstrated the nuclear localization of PI in all the samples (Figure 2A(i) upper panel) indicating the cells that had moved into the late apoptotic/necrotic stages normally in all samples. Cells positive for AV green fluorescence were noted prominently on the cell surface for early and late apoptotic cells in both the control (Figure 2A(ia)) and cisplatin (Figure 2A(ib))-treated samples, while there was an overall reduction in the green fluorescence in samples treated with the nanoparticles, as demonstrated by the representative images in Figure 2A(ic–f)), thus signifying the cellular health-modifying properties of CeO_2_ NPs in terms of decreased apoptosis. These findings were confirmed by the flow cytometry, which showed that there was an overall increase in the healthy cell population, with a corresponding decrease in apoptotic and dead cells (Figure 2A(ii)). This increase in the healthy cell population was highly significant for the samples treated with 100 and 200 µg/mL CeO_2_ NPs, while a significant concentration-dependent decrease in late apoptotic cells was noted for the samples treated with 50, 100, and 200 µg/mL CeO_2_ NPs (Figure S2i). The number of dead cells in most of the NP-treated samples was near to, or less than, that of the untreated control samples; however, this decrease was not significant. None of the treatments with 10 µM cisplatin resulted in any statistically significant changes in the cell populations. This was expected, since cisplatin at the physiological dose of 10 µM, a concentration most commonly reaching the tissues in clinical treatments, is considered ineffective against MDA MB231 cell viability [62,63], and at lower concentrations has even been shown to paradoxically increase the overall cell proliferation [64], as observed in the cisplatin-treated healthy population (Figure S2i). We continued to use a 10 µM cisplatin concentration in our further experiments to compare the results following irradiation with those of nanoparticle-treated irradiated samples.

The results of the rhodamine 123 experiments in unirradiated samples showed a dramatic decrease in ROS levels in proportion to the CeO_2_ nanoparticle treatment concentrations, and this decrease was highly significant when compared to that of the untreated samples (Figure 2B and Figure S2ii). This was confirmed in previous studies [43] and currently forms the basis of our irradiation experiments. Cerium oxide nanoparticles have oxygen vacancies in their crystal lattice and are redox-active owing to their special ability to engage in a cyclic reaction on their surface that is auto-regenerated between two electronic forms: Ce^3+^ ⇌ Ce^4+^ [65]. DHR 123 in labeled cells is oxidized to green-fluorescent rhodamine 123 by peroxynitrite anion [ONOO]^−^ and hydrogen peroxide anion [HOO]^−^ species in cells, which acts as an indicator of the levels of reactive oxygen species in the cells.

We then went on to look at the effect of different absorbed doses of photon radiation on the health of MDA MB231 cells that were not treated or treated with varying concentrations of CeO_2_ nanoparticles (Figure 3). The cells treated with 10µM cisplatin continued to serve as positive controls in the irradiation experiments. The gated flow cytometry results showed that the healthy population steadily increased in a nanoparticle-concentration-dependent manner for samples irradiated with 0.1 Gy (Figure 3A, top panel, and 3B(i)), with the 200 µg/mL treatment group showing the most drastic increase when compared to the untreated irradiated control (Figure 3B(i) FITC-A^−^, DsRed-A^+^). There was an overall corresponding decrease in the late apoptotic and necrotic population, with the 200 µg/mL once again showing a dramatic decrease in the former population when compared to that of the untreated irradiated cells (Figure 3B(i) FITC-A^+^, DsRed-A^+^). Unlike what was seen in the unirradiated samples, cisplatin-treated cells that were irradiated with 0.1 Gy showed an apparent overall decrease in the healthy population, with corresponding increases in late apoptotic and necrotic cells (Figure 3A (top panel), 3B(i)). The increase in the healthy population of the nanoparticle-treated samples was more significant when compared with that of the cisplatin-treated positive control group (Figure 3B(i) FITC-A^−^, DsRed-A^−^). Moreover, when comparing the decrease in the late apoptotic and necrotic populations to the cisplatin-treated positive controls, the 100 and 200 µg/mL nanoparticle-treated samples showed the most significant decrease. The early apoptotic population did not display any remarkable changes for any of the treatment groups.

We then looked at the effect of 1 Gy on the nanoparticle-treated cells at various concentrations (Figure 3A, middle panel, and Figure 3B(ii)). Overall, the healthy population increased for the samples treated with nanoparticles, most significantly for the highest concentration, when compared to the untreated irradiated negative controls. The late apoptotic and necrotic population also showed a corresponding decrease following the nanoparticle treatment in a concentration-dependent manner, with the 200 µg/mL treatment showing the most significant effect when compared to the negative controls. In these experiments, too, cisplatin-treated irradiated cells served as a positive control. Though not significant, there was an overall decrease in the number of healthy cells, with a corresponding increase in early and late apoptotic populations following cisplatin-coupled radiation treatment when compared to the irradiated negative control. When comparing to this cisplatin-treated irradiated positive control group, the decrease in the late apoptotic population was quite significant across all concentrations of the nanoparticle-treated samples, except for the lowest treatment (25 µg/mL). The changes noted in the early apoptotic populations were unremarkable.

The cells irradiated with 10 Gy showed an overall increase in healthy population with all but the lowest tested nanoparticle concentration, and the most significant amelioration of cellular health when compared to the untreated irradiated negative control was once again observed at the highest treatment concentration (200 µg/mL). (Figure 3A, lower panel, and Figure 3B(iii)). An overall decrease in the late apoptotic and dead cells was also noted in a concentration-dependent manner for the nanoparticle-treated cells when compared to the negative controls, with the most significant reduction being observed for late apoptosis in the 200 µg/mL treatment group (*p* < 0.005). Cisplatin-induced changes were unremarkable or meagre at this radiation dose when compared to those of the untreated irradiated controls; however, when compared to the former, the increase in healthy cells and the corresponding decrease in late apoptotic cells appeared to be significant at the highest treatment concentrations of the nanoparticles at 10 Gy. Even though the 200 µg/mL nanoparticle treatment consistently brought about the most dramatic effects to the various examined cell populations, including a betterment of cellular health, this amelioration of cellular health was seen significantly tapering off with higher radiation doses (Figure S3, 200 µg/mL). Furthermore, looking at the cisplatin-induced responses across the various radiation treatments, both cisplatin and radiation seemed to act synergistically to reduce the overall cellular health at all radiation doses. Even though this reduction of cisplatin samples was not very intense (Figure S3, 0 µg/mL + Cisp), it enabled an effective comparison and demonstration of the significance of the changes to cellular health brought about by the nanoparticles.

To explore the role of nanoparticles in modulating reactive oxygen species in the context of cellular health, we gated for rhodamine 123 expression in cells not labeled or labeled with DHR123 following treatment with or without nanoparticles at various concentrations and radiation absorbed doses (Figure 4). A significant amount of ROS was observed in samples not treated with nanoparticles following irradiation in comparison to the unirradiated samples. However, there was a drastic reduction in ROS levels across all nanoparticle-treated samples and at all radiation absorbed doses (*p* < 0.0001) (Figure 4B(i)). The amount of radiation-induced ROS was almost at the background levels at the highest treatment concentration of 200 µg/mL, and this was consistently observed at all radiation doses (Figure 4A). When correlating with the results from the cellular health experiments, an increasingly healthy population of cells was observed in conjunction with a reduction in reactive oxygen species in proportion to the nanoparticle concentration in the cells. Moreover, at the highest treatment concentrations of the nanoparticles, and despite ROS being reduced to almost background levels, the amelioration in cellular health was often found to be significantly higher than that of the untreated controls. This effect was much more pronounced at the lower radiation absorbed dose (0.1 Gy) than at the higher doses (1 and 10 Gy) (Figure 3). Interestingly, in the control cells not treated with nanoparticles, a significant ROS production was observed, along with an increase in the healthy population proportionate to the increasing radiation doses, though this effect appeared to taper off at higher doses (Figure 3A, first column, and Figure 4A, second column). The significance of this paradoxical finding is unclear, but previous studies have pointed to the role of reactive oxygen species as critical signaling molecules in cell proliferation and survival [66]. This may also point to yet another mechanism, rather than absolute levels of ROS for mediating cellular health, which warrants further investigation.

Our results contrast with those of two recent studies on human MCF-7 breast cancer cells, which reported no radioprotective effects by CeO_2_ nanoparticles at any radiation dose or treatment concentration [67,68]. A previous study in 2007 also reported that when treated with 5000 nM nanoceria in combination with 10 Gy radiation, MCF-7 breast cancer cells were not protected from radiation-induced cell death [69]. However, all these studies report a selective radioprotection by CeO2 nanoparticles in normal cells or other cell types. We believe this may be attributable to local factors such as pH differences, cell compartmental localization differences, and other modifying factors that may be cell-type-specific.

In conclusion, we have shown unequivocally for the first time that unmodified CeO_2_ nanoparticles are taken up into MDA MB231 cells using macropinocytosis and are aggregated in large macropinosomes in a concentration-dependent manner. These nanoparticles decreased cellular apoptosis with a corresponding dramatic amelioration of cellular health both in the unirradiated and irradiated populations at all absorbed doses of ionizing radiation. These changes in cellular health were nanoparticle-concentration-dependent and resulted in a simultaneous, very large decrease in reactive oxygen species in both unirradiated and irradiated populations at all absorbed radiation doses. Moreover, the most dramatic effects following irradiation were observed at the highest nanoparticle treatment concentrations, in which the additional ROS generated in irradiated cells was reduced to background levels, with the cells having better health compared to untreated unirradiated populations. These studies serve as a caution for the use of CeO_2_ nanoparticles for cancer treatment without or with radiotherapy, but its unique radioprotective effects may afford significant opportunities for exploring its use in the context of mitigating the bystander effects of radiation in normal cells.

## Figures and Tables

**Table 1:** Flow cytometry gated profiles of viable (AV^−^/PI^−^), early apoptotic (AV^+^/PI^−^), apoptotic and necrotic (AV^+^/PI^+^), and already dead (AV^−^/PI^+^) cells treated with varying concentrations of CeO_2_ NP, or Cisplatin.

Treatment	AV^−^/PI^−^	AV^+^/PI^−^	AV^+^/PI^+^	AV^−^/PI^+^
0 µg mL^−1^ CeO_2_	37%	21%	36%	7%
25 µg mL^−1^ CeO_2_	47%	18%	28%	7%
50 µg mL^−1^ CeO_2_	41%	42%	16%	1%
100 µg mL^−1^ CeO_2_	69%	9%	13%	9%
200 µg mL^−1^ CeO_2_	72%	17%	9%	1%
Cisplatin	58%	11%	20%	11%

**Table 2:** Flow cytometry gated profiles of viable (AV^−^/PI^−^), early apoptotic (AV^+^/PI^−^), apoptotic and necrotic (AV^+^/PI^+^), and already dead (AV^−^/PI^+^) cells treated with varying concentrations of CeO_2_ NP, or Cisplatin and irradiated with 0.1 Gy ionizing radiation.

Treatment	AV^−^/PI^−^	AV^+^/PI^−^	AV^+^/PI^+^	AV^−^/PI^+^
0 µg mL^−1^ CeO_2_	57%	10%	23%	10%
0 µg mL^−1^ + Cisplatin	43%	13%	25%	19%
25 µg mL^−1^ CeO_2_	66%	5%	17%	12%
50 µg mL^−1^ CeO_2_	52%	13%	26%	9%
100 µg mL^−1^ CeO_2_	85%	12%	18%	6%
200 µg mL^−1^ CeO_2_	84%	12%	4%	1%

**Table 3:** Flow cytometry gated profiles of viable (AV^−^/PI^−^), early apoptotic (AV^+^/PI^−^), apoptotic and necrotic (AV^+^/PI^+^), and already dead (AV^−^/PI^+^) cells treated with varying concentrations of CeO_2_ NP, or Cisplatin and irradiated with 1 Gy ionizing radiation.

Treatment	AV^−^/PI^−^	AV^+^/PI^−^	AV^+^/PI^+^	AV^−^/PI^+^
0 µg mL^−1^ CeO_2_	68%	6%	18%	7%
0 µg mL^−1^ + Cisplatin	54%	8%	28%	9%
25 µg mL^−1^ CeO_2_	56%	8%	28%	7%
50 µg mL^−1^ CeO_2_	70%	11%	14%	5%
100 µg mL^−1^ CeO_2_	70%	10%	15%	5%
200 µg mL^−1^ CeO_2_	73%	16%	10%	1%

**Table 4:** Flow cytometry gated profiles of viable (AV^−^/PI^−^), early apoptotic (AV^+^/PI^−^), apoptotic and necrotic (AV^+^/PI^+^), and already dead (AV^−^/PI^+^) cells treated with varying concentrations of CeO_2_ NP, or Cisplatin and irradiated with 10 Gy ionizing radiation.

Treatment	AV^−^/PI^−^	AV^+^/PI^−^	AV^+^/PI^+^	AV^−^/PI^+^
0 µg mL^−1^ CeO_2_	65%	6%	22%	7%
0 µg mL^−1^ + Cisplatin	56%	10%	26%	8%
25 µg mL^−1^ CeO_2_	59%	6%	23%	12%
50 µg mL^−1^ CeO_2_	64%	10%	18%	8%
100 µg mL^−1^ CeO_2_	64%	11%	21%	4%
200 µg mL^−1^ CeO_2_	73%	14%	11%	2%

**Table 5:** Flow cytometry gated profiles of unlabeled or DHR123 labeled cells treated with varying concentrations of CeO_2_ NP and irradiated with 0.1, 1, or 10 Gy ionizing radiation.

Treatment	0.1 Gy	1 Gy	10 Gy
0 µg mL^−1^ CeO_2_ (unlabeled)	0%	0%	0%
0 µg mL^−1^ CeO_2_	33%	36%	36%
25 µg mL^−1^ CeO_2_	12%	12%	20%
50 µg mL^−1^ CeO_2_	6%	7%	14%
100 µg mL^−1^ CeO_2_	2%	3%	5%
200 µg mL^−1^ CeO_2_	1%	1%	1%

## Data Availability

The data used to support the findings of this study are included within the article and as supplementary files.

## Supplementary Materials

The following are available online at https://www.mdpi.com/article/10.3390/biology10111148/s1, Figure S1: Transmission electron microscopy image of cerium oxide nanoparticles in suspension, Figure S2: Interpopulation AOVA of cellular health and ROS in unirradiated samples, Figure S3: Intrapopulation ANOVA of cellular health of irradiated samples at various nanoceria concentrations, Figure S4: ANOVA statistical analyses data files.
