# Supplementary figures and images for: Internalized Nanoceria Modify the Radiation-Sensitivity Profile of MDA MB231 Breast Carcinoma Cells
Source: Biology (Basel). 2021 Nov 8;10(11):1148. doi: 10.3390/biology10111148 (PMC8614948; doi:10.3390/biology10111148)

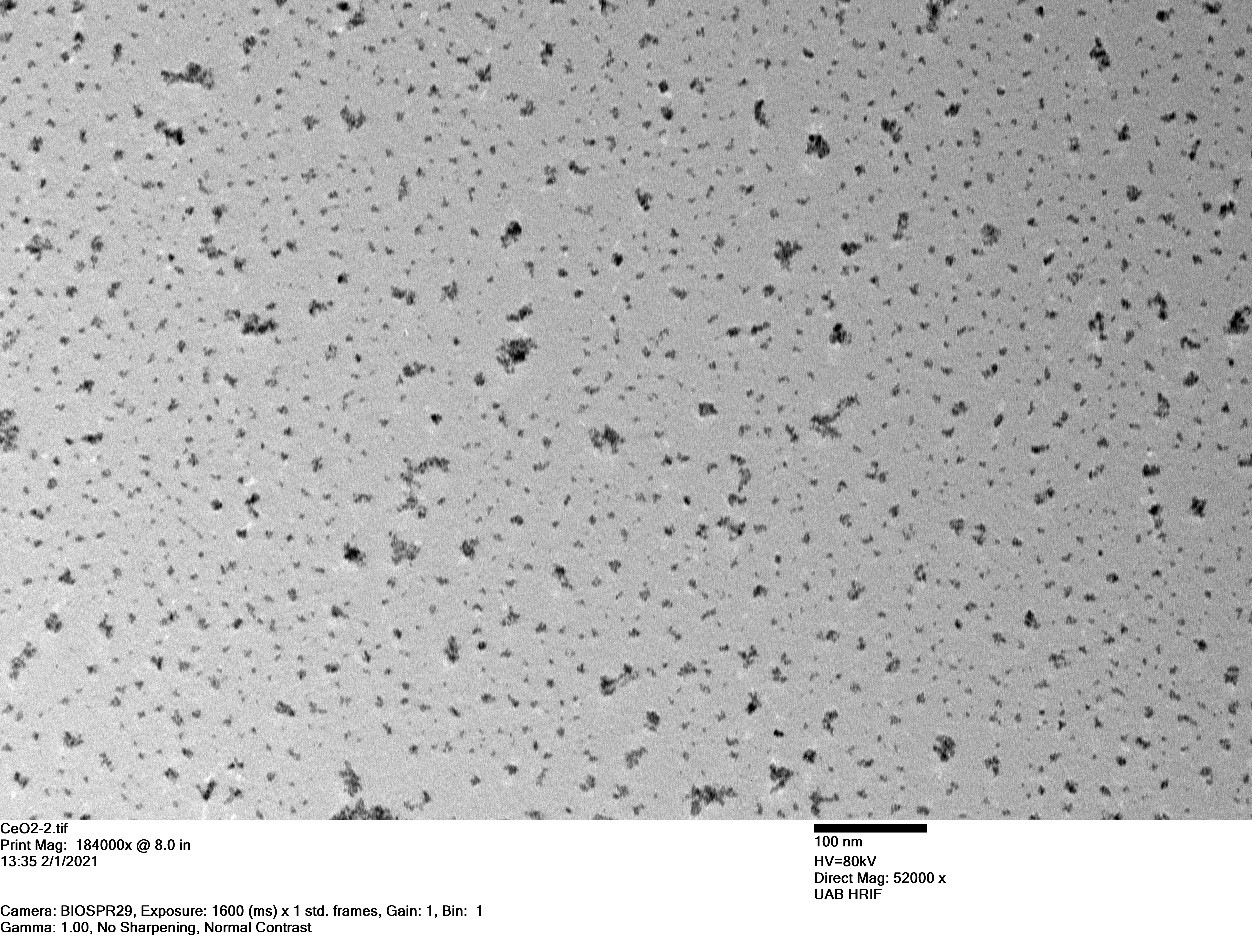

Supplement: Supplementary file 1 [file biology-10-01148-s001.zip › Fig. S1.tif]

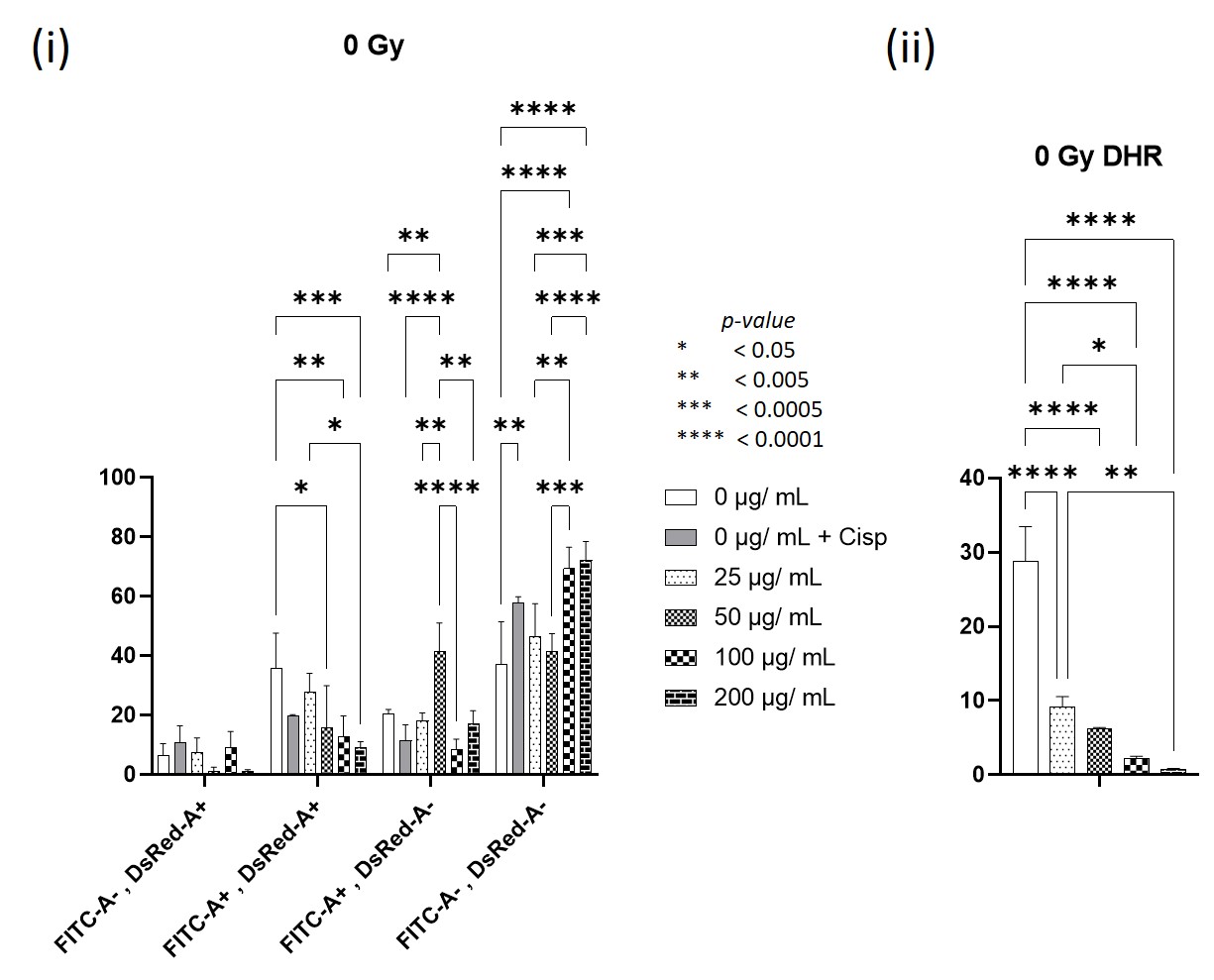

Supplement: Supplementary file 1 [file biology-10-01148-s001.zip › Fig. S2.jpg]

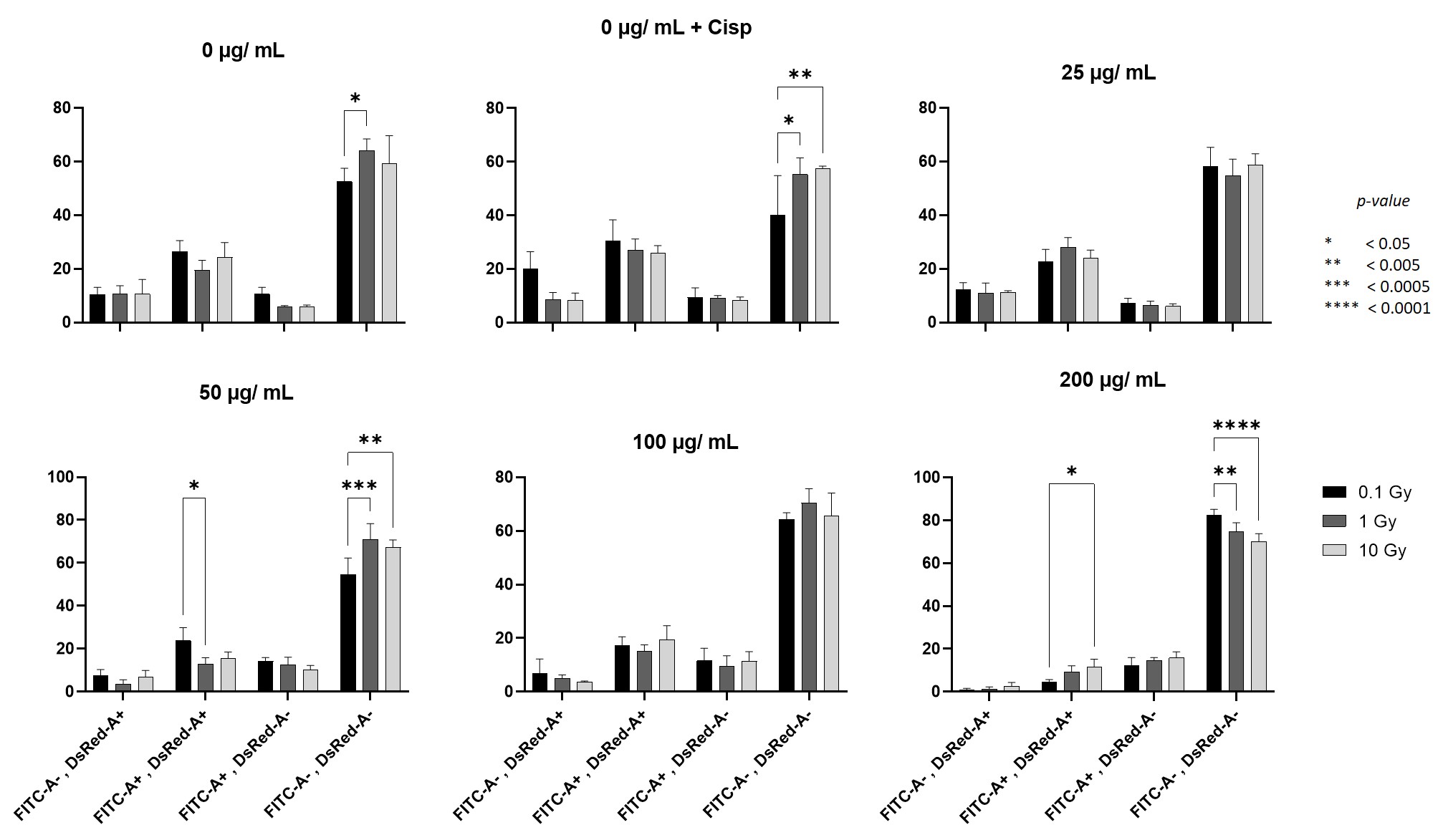

Supplement: Supplementary file 1 [file biology-10-01148-s001.zip › Fig. S3.jpg]
